# Macrophagic activation syndrome revealing disseminated multifocal tuberculosis: A case report of a rare clinical situation

**DOI:** 10.1016/j.amsu.2022.103487

**Published:** 2022-03-09

**Authors:** Sara Gartini, Youssef Bougrini, Meriem Rhazari, Jamal Eddine Bourkadi

**Affiliations:** a Pneumology Department, Mohammed VI University Hospital, Oujda, Morocco; b Mohammed First University Oujda, Faculty of Medicine and Pharmacy Oujda, Oujda, Morocco; c Pneumology Department, Moulay Ismail Military Hospital, Meknes, Morocco; d Pneumology Department, Moulay Youssef Hospital – Ibn Sina University Hospital, Rabat, Morocco

**Keywords:** Macrophagic activation syndrome, Disseminated multifocal tuberculosis, Pancytopenia

## Abstract

**Introduction:**

Macrophage activation syndrome (MAS) is a rare but serious entity, sometimes triggered by an infectious agent, in particular by certain viruses or mycobacteria. Forms of tuberculosis complicated by MAS frequently occur in immunocompromised subjects. The disease is often disseminated, severe, and has a poor prognosis.

**Case presentation:**

We report a case of MAS complicating disseminated tuberculosis and revealing HIV in a previously healthy young patient.

**Discussion & conclusion:**

The management of macrophage activation syndrome related to tuberculosis is complex and not codified. On the one hand, the immunosuppressive treatment allows to slow down the macrophage activation syndrome and obtain a correction of cytopenia. On the other hand, there is a risk of aggravating tuberculosis by increasing the patient's immunodepression.

## Highlights

• Macrophagic activation syndrome (MAS) represents a rare and serious entity.
• Forms of tuberculosis complicated by MAS frequently occur in immunocompromised subjects.
• The clinical finding is aspecific and the search for medullary hemophagocytosis is part of the work-up.
• The management of MAS related to tuberculosis is complex and not codified.

## 1 Introduction

Macrophage activation syndrome (MAS) is a rare but serious entity, sometimes triggered by an infectious agent, in particular by certain viruses or mycobacteria. Tuberculosis complicated by MAS frequently occurs in immunocompromised subjects. It is disseminated in most cases, with a severe prognosis [1]. This case report presents the observation of a young immunocompromised patient (HIV seropositive) with disseminated tuberculosis complicated by MAS.

## 2 Case presentation

A 35-year-old man, with no personal or familial history of tuberculosis. He has no médical or surgical history. The patient was initially hospitalized in the internal medicine department for the management of an anemic syndrome (asthenia, dizziness, dyspnea), associated with right hypochondrium pain and a dry cough evolving for a month in a context of fever, anorexia and weight loss of 10 kg in one month. The clinical examination found a patient in fairly good general condition, pale. The respiratory examination was normal with an oxygen saturation of 96% in ambient air, the abdominal examination showed a dullness in the right hypochondrium with hepatosplenomegaly. The biological workup showed pancytopenia: anemia at 9.6 g/dl normochromic normocytic, Leukopenia at 1500/μL with lymphopenia at 180/μL and thrombocytopenia at 54000/μL, mild liver cytolysis (AST 120 IU/L [N < 35], ALAT 142 IU/L [N < 55]), cholestasis (PAL 290 IU/L [N < 120], GGT 269 [N < 64], hyperferritinemia at 11,000 ng/ml; hypertriglyceridemia at 2.2 g/L, LDH: 432 U/L, negative direct Coombs test, fibrinogen level at 1.8 g/L [N 2.0–4.0]. In addition, HIV serology was positive. The other viral serologies: syphilis, Epstein-Barr virus, cytomegalovirus, HCV, and HBV were negative.

The immune workup (antinuclear antibodies, ANCA, protein electrophoresis with weighted immunoglobulin assay) showed only a polyclonal increase in immunoglobulins with IgG at 26 g/L (N < 18.5) IgM at 6.06 (N < 2.4).

The febrile pancytopenia with splenomegaly led to a myelogram which revealed a multilineage dysmyelopoiesis. The osteo-medullary biopsy showed granulomatous myelitis with caseous necrosis in favor of hematopoietic tuberculosis.

**Fig. 1:**
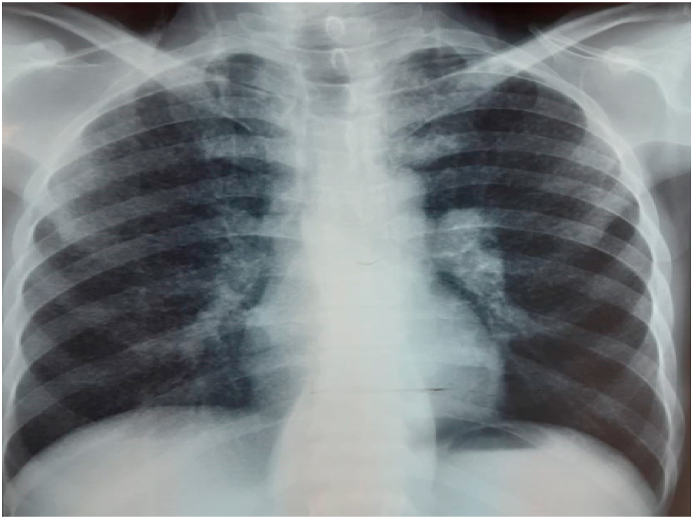
Chest X-ray of our patient: diffuse micronodular opacities in both lung fields giving a Milliary appearance.

The chest X-ray showed diffuse micronodular opacities with homogeneous millet grain distribution on both sides, suggesting a miliary tuberculous aspect (Fig. 1).

The Xpert MTB/RIF test for BK in the sputum came back positive, rifampicin resistance was not detected.

**Fig. 2:**
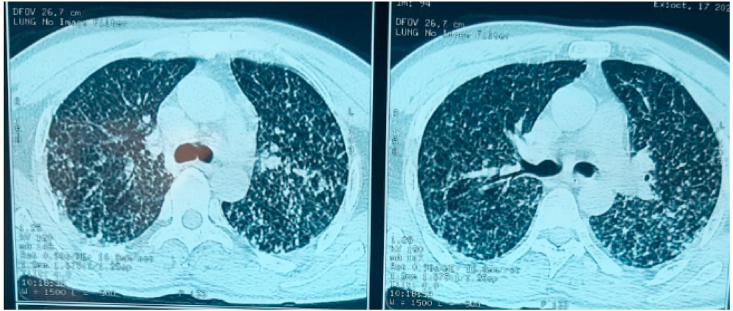
Chest CT scan of our patient showing multiple bilateral parenchymal micronodules, diffused, evoking a miliary tuberculosis.

A thoracic and abdominopelvic CT scan was performed, showing multiple bilateral parenchymal micronodules on the thoracic level, diffused and giving a miliary appearance. On the abdominopelvic level, multiple bilateral iliac and mesenteric adenopathies with necrotic centers, hepatomegaly, and a globular spleen with multiple hypodense lesions were noted, suggesting a tubercular origin (Fig. 2).

**Fig. 3:**
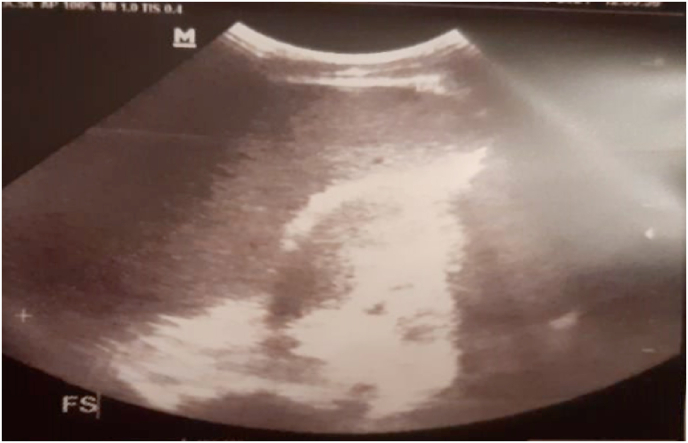
Abdominal ultrasound: showing splenomegaly measuring 14 cm.

**Fig. 4:**
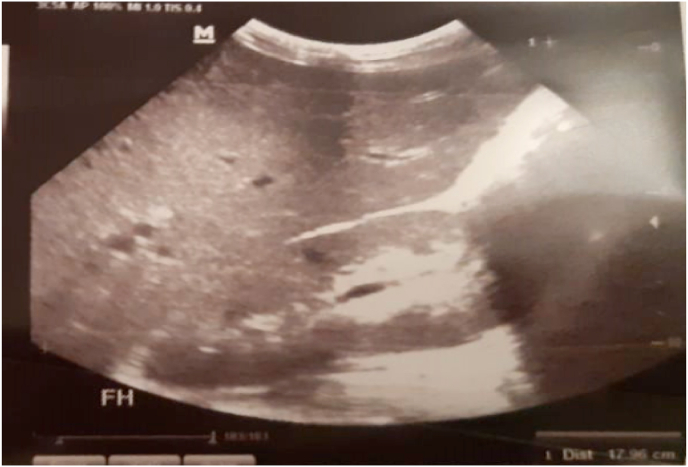
Abdominal ultrasound: showing hepatomegaly with regular contours and homogeneous structure.

Abdominal ultrasound showed an enlarged liver with regular contours and a homogeneous echostructure. An enlarged spleen of homogeneous echostructure measuring 14 cm (Fig. 3, Fig. 4).

MAS was retained on clinical and biological criteria.

The diagnosis of multifocal tuberculosis revealed by a macrophage activation syndrome in a patient immunocompromised with HIV+. An anti bacillary treatment was initiated (two months of a quadritherapy “intensive phase” associating Rifampicin, Isoniazid, Ethambutol and Pyrazinamide, and seven months of a bitherapy “continuation phase” associating Rifampicin and Isoniazid) for a total duration of nine months with supplementation in vitamin B6 at a preventive dose of 25 mg/dr and cotrimoxazole prophylaxis. With a clinical and biological improvement (correction of pancytopenia). Antiretroviral therapy was started at the fourth week after the beginning of anti-tuberculosis treatment. The patient presented two weeks after the beginning of the anti bacillary treatment a worsening of the hepatic cytolysis (ASAT 540 UI/L [N < 35], ALAT 346 UI/L [N < 55]),a cholestasis (PAL 391 UI/L [N < 120], GGT 367 [N < 64]. The anti-bacillary treatment was stopped and substituted by a non hepatotoxic treatment (Kanamycin, Ethambutol, Levofloxacin) because of the severe form of tuberculosis.

The evolution was favorable. Fever and night sweats disappeared in five days. The blood count and liver biology were normalized in ten days. Antibacillary treatment with ERIPK4 was reintroduced over three days with good tolerance. The patient's general condition improved more slowly with a 4 kg gain in one month. The respiratory evolution was favorable with a progressive regression of cough and dyspnea at the end of the second month.

## 3 Discussion

MAS, also known as a hemophagocytic syndrome, is defined by a set of clinical and biological signs related to the proliferation and histiocytic activation of the reticuloendothelial system, leading to anarchic phagocytosis of figurative blood elements and marrow precursors [1].

MAS can be either:

- Primitive (or familial), most often found in children under two years such as familial lymphohistiocytosis.
- Secondary: to hematological pathologies, in particular T-cell lymphomas, autoimmune diseases, or infections including tuberculosis [2,3], as in the present case. MAS can be associated with multiple viral (EBV, HIV, CMV), bacterial (*Salmonella typhi*) and parasitic (Leishmania sp., Toxoplasma gondii) causes.

The physiopathology is still poorly understood: possible hypersecretion of cytokines (including TNF-alpha, IFN-gamma, IL-6) by activated macrophages, responsible for the clinical and biological manifestations.

The clinical presentation is nonspecific: a revealing high fever (39–40 °C) is constant as well as an altered general condition, jaundice, and hepatosplenomegaly. Adenopathies are also frequent, neurological signs (localization signs, convulsions) are rarer. Biology reveals pancytopenia (thrombocytopenia is constant; anemia and leukopenia are inconstant), hypertriglyceridemia due to inhibition of lipoprotein lipase by TNF, hepatic cytolysis, and hyperferritinemia (secreted by activated macrophages). It also represents a good marker of disease activity and therapeutic response and can be used as a prognostic indicator [4]. Our patient had all these criteria.

The search for bone marrow hemophagocytosis is part of the work-up, but it is neither sensitive (70–83%) nor specific (60%) [5,6] and its presence is therefore neither sufficient nor necessary for the diagnosis of MAS. The prevalence of hemophagocytosis varies between 25 and 100% of MAS depending on the study [7] and can sometimes be delayed and appear after the initiation of treatment, justifying the repetition of blood tests.

An osteo-medullar biopsy may often underestimate active hemophagocytosis and appears to be less effective than myelograms.

The association of hemophagocytic syndrome with tuberculosis remains very rare. The subject has been discussed for several years: in 1993, Cassim et al. [8] reported what they called a reactive histiocytic hemophagocytosis associated with disseminated tuberculosis; in 1995 and 1996, Quinquandon et al. [9] and Undar et al. [10] respectively reported two cases of hemophagocytosis associated with tuberculosis; in 1998, Francois et al. attempted to link pancytopenia of tuberculosis to hemophagocytosis. Hemophagocytic syndrome is mainly found in extrapulmonary tuberculosis. Brastianos et al., in 2006, were able to collect retrospectively only 37 cases in the literature, proving the rarity of this association. Of these 37 patients, 29 had extrapulmonary tuberculosis and 27 had involvement of a hematopoietic organ, as was the case with our patient. Ten patients did not have a hematopoietic location but had a macrophage activation syndrome. For three patients, data regarding the site of tuberculosis are missing [11]. Nevertheless, one may wonder whether the cytopenias classically observed in cases of tuberculosis of the hematopoietic organs are not secondary to a mechanism of hemophagocytosis, since this cytological abnormality may be unrecognized or not very visible on bone marrow investigations. However, not all hematopoietic localizations of tuberculosis are associated with a macrophage activation syndrome.

Indeed, splenic tuberculosis, a rare entity (68 cases reported in the literature in the last ten years [12]), is often associated with synchronous liver involvement and is usually not accompanied by hemophagocytosis [8].

A recent review of the literature [13] identified only 37 published cases of tuberculosis associated with MAS: half of the patients were immunocompromised (renal transplant with immunosuppressive treatment, cancer, HIV). The same clinical signs were present (fever, hepatosplenomegaly). Eighty percent of the cases had disseminated tuberculosis. Mortality was approximately 50%. All patients who did not receive anti-tuberculosis treatment died. Immunosuppressive therapies in addition to anti-tuberculosis treatment, such as high-dose corticosteroids, plasma exchange, intravenous immunoglobulin, or splenectomy, had no net benefit on survival.

According to the HAS [14], the treatment with intravenous immunoglobulin for MAS associated with infectious cause is not recommended due to lack of evidence, but this treatment is sometimes practiced with some recorded success [15]. The goal of the treatment of hemophagocytosis is to attenuate the inflammatory response. At present, due to the rarity of MAS and the heterogeneity of its causes, no randomized controlled trials exist to propose precise therapeutic recommendations. Among the causes of post-infectious MAS [16], only MAS secondary to EBV has a reference treatment which is etoposide. However, most authors agree on the use of corticosteroids at a dose of 1 mg/kg per day. Thus, there is currently no specific recommendation for the management of MAS of tuberculosis origin. Once again, early anti-bacillary treatment is the most important.

## 4 Conclusion

Macrophage activation syndrome is a rare but serious entity, sometimes triggered by an infectious agent, notably tuberculosis. The management of MAS related to tuberculosis is complex and not codified. The assessment of the benefit-risk ratio is difficult, due to the risk of immunosuppression linked to the use of corticosteroids or immunosuppressive drugs, with possible aggravation of tuberculosis. On the one hand, immunosuppressive treatment can slow down the macrophage activation syndrome and correct cytopenias. On the other hand, there is a risk of aggravating tuberculosis by reducing the patient's immunocompetence. Close monitoring of the patient is essential in all cases to adapt the therapy as well as possible.

## Provenance and peer review

Not commissioned, externally peer reviewed.

## Ethical approval

No ethical approval necessary.

## Sources of funding

The author(s) received no financial support for the research, authorship and/or publication of this article.

## Registration of research studies

Our paper is a case report; no registration was done for it.

## Consent

Written informed consent was obtained from the patient for publication of this case report and accompanying images. A copy of the written consent is available for review by the Editor-in-Chief of this journal on request.

## Author contribution

Sara Gartini: Writing, review and editing of the manuscript.

Youssef Bougrini, Meriem Rhazari: Contributed for diagnose and treatment of the patient.

Jamal Eddine Bourkadi: Supervised the writing of manuscript.

## Guarantor

Gartini Sara.

## Declaration of competing interest

The authors declared no potential conflicts of interests with respect to research, authorship and/or publication of the article.
